# Distribution of elongation factor-1α in larval tissues of the fall armyworm, Spodoptera frugiperda

**DOI:** 10.1673/2006_06_33.1

**Published:** 2006-10-25

**Authors:** Javad Habibi, Cynthia L. Goodman, Melissa K. Stuart

**Affiliations:** 1 Department of Internal Medicine, University of Missouri, Columbia MO; 2 U.S. Department of Agriculture, Agricultural Research Service, Biological Control of Insects Research Laboratory, Columbia, MO; 3 Department of Microbiology/Immunology, A.T. Still University, Kirksville, MO

**Keywords:** lepidopteran, translation, apoptosis, monoclonal antibody, enzyme-linked immunosorbent assay (ELISA), immunofluorescence microscopy

## Abstract

Elongation factor-1α (EF-1α) promotes the delivery of aminoacyl-tRNA to the acceptor site of the ribosome during protein synthesis. The enzyme has a number of additional functions, including regulation of apoptosis and interaction with the cytoskeleton. We determined the distribution of EF-1α in larval tissues of the fall armyworm, Spodoptera frugiperda, with a monoclonal antibody generated to EF-1α from Sf21 cells, a cell line developed from ovarian tissue of S. frugiperda. Enzyme-linked immunosorbent assay showed that EF-1α comprised 1.9–9.9 % of the total protein within the tissues that were examined, which included fat body, Malpighian tubules, midgut, muscle, salivary glands, trachea, and ventral nerve cord. To a certain extent, EF-1α concentrations reflected the expected metabolic activity level of each of the represented tissues. Closer examination by immunofluorescence microscopy revealed that EF-1α concentrations varied among different cell types within a given tissue, i.e. midgut columnar epithelial cells yielded strong signals, while goblet cells failed to react with the EF-1α -specific antibody.

## Introduction

Elongation factor-1 alpha (EF-1α) is an enzyme that promotes GTP-dependent binding of aminoacyl-tRNA to the acceptor site on ribosomes during protein synthesis (Negrutskii and El’skaya 1998). The enzyme has a number of additional functions, including actin binding and bundling (Murray et al. 1996; Dharmawardhane et al. 1991), microtubule severing (Shiina et al. 1994), and regulation of apoptosis (Ruest et al. 2002; Duttaroy et al. 1998). Multiple forms of EF-1α encoded by paralogous genes are found in many eukaryotic taxa, including yeasts (Sundstrom et al. 1991; Linz and Sypherd 1987), ciliates (Bergemann et al. 1996), plants (Axelos et al. 1989), amphibians (Djé et al. 1990), mammals (Kristensen et al. 1998; Lee et al. 1992), and arthropods (Hovemann et al. 1988). The isoforms display stage- and tissue-specific expression patterns. For example, three variants of EF-1α are expressed in the African clawed frog, Xenopus laevis: 42Sp50 is expressed exclusively in immature oocytes and appears to store tRNAs for later use in oogenesis and embryogenesis; EF-1αO is expressed mainly in oocytes, transiently in early embryogenesis, and not at all in somatic cells after neurulation; and EF-1αS is expressed at low levels in oocytes but actively in somatic cells (Djé et al. 1990). eEF-1αO mRNA is also present in spermatogonia and spermatocytes of adult testis (Abdallah et al. 1991). In mammals, there are two actively translated EF-1α genes, which encode the eEF1A1 and eEF1A2 isoforms. eEF1A1 is expressed ubiquitously in all tissues and developmental stages, while expression of eEF1A2 is restricted to heart, brain, and skeletal muscle of adults (Knudsen et al. 1993; Lee et al. 1992).

Among the holometabolous dipterans (Hovemann et al. 1988), hymenopterans (Danforth and Ji 1998), and coleopterans (Jordal 2002) two functional EF-1α genes, called F1 and F2 are expressed. In Drosophila melanogaster, F1 mRNA is present throughout development, while F2 mRNA is highly expressed in the pupal stage and to a lesser extent in third instars and adults (Hovemann et al. 1988). In contrast to the other holometabolous insects, lepidopterans reportedly have just one copy of the EF-1α gene (Mitchell et al. 1997; Cho et al. 1995), but even from a single gene multiple forms of the enzyme can be generated by post-translational modifications. In many eukaryotes, EF-1α is methylated (Hiatt et al. 1982; Sherman and Sypherd 1989), phosphorylated (Izawa et al. 2000; Venema et al. 1991), and modified by the addition of glycerylphosphorylethanolamine groups (Dever et al. 1989; Whiteheart et al. 1989). Phosphorylation of EF-1α increases the overall rate of protein elongation (Chang and Traugh 1998; Venema et al. 1991), but the significance of the additional modifications in multicellular organisms has not been determined with certainty (Cavallius et al. 1997; Whiteheart et al. 1989).

In a previous study, a monoclonal antibody (Mab 7D6) was generated to EF-1α from Sf21 cells (Stuart and Chamberlain 2003), a cell line established from ovarian tissue of the fall armyworm Spodoptera frugiperda (Lepidoptera: Noctuidae) (Vaughn et al. 1977). The Mab inhibited in vitro translation by 75% when added to lysates of Sf21 cells at a 2:1 antibody:EF-1α ratio. Mab 7D6 also recognized EF-1α in both neuronal and ovarian cell lines established from Heliothis virescens, but failed to react with an embryonic cell line derived from Trichoplusia ni; both are Noctuidae. Western blot analysis of whole-body insect homogenates showed that the form of EF-1α recognized by Mab 7D6 was not unique to established cell lines. Instead, the antibody also avidly recognized EF-1α in eggs and all larval stages of S. frugiperda, but the tissue distribution of the enzyme was not determined. Studies conducted on rabbit tissues (Slobin 1980) and cultured cells (Sanders et al. 1992) indicate that concentrations of EF-1α vary widely depending on the tissue or cell source being examined. For this reason, we hypothesized that EF-1α would show a differential expression pattern among the various tissues of S. frugiperda larva probed with Mab 7D6. This hypothesis is tested herein.

## Materials and Methods

### Monoclonal antibody 7D6 (Mab 7D6)

The production and characterization of Mab 7D6 generated to S. frugiperda EF-1α has been described previously (Stuart and Chamberlain 2003). Mab 7D6 immunoprecipitates a single protein of 53 kDa, identified as EF-1α by Edman degradation, from homogenates of Sf21 cells. To concentrate the antibody and exchange protein-free culture medium for phosphate-buffered saline (PBS) (137 mM NaCl, 2.7 mM KCl, 1.5 mM KH_2_PO_4_, 8.1 mM Na_2_HPO_4_, pH 7.4), hybridoma supernates containing Mab 7D6 were subjected to ultrafiltration through a Biomax-30 membrane (Fisher Scientific, www.fishersci.com). Aliquots of stock antibody (0.83 mg/ml) were stored at −80ºC until used in the immunoassays described below. The antibody concentration was determined using a Coomassie Blue dye-binding method (Bio-Rad Laboratories, www.bio-rad.com), with bovine gamma globulin serving as the standard.

### Measurement of EF-1α concentrations in larval tissues by enzyme-linked immunosorbent assay (ELISA)

 S. frugiperda larvae were purchased from Bio-Serv (www.bio-serv.com) and reared on artificial diet (F9772, Bio-Serv) at 27ºC with a 16 h:8 h light:dark cycle until their fifth stadium. For dissections, after the initial incision, midgut tissues were removed and the body cavity was thoroughly rinsed with PBS (137 mM NaCl, 4 mM KCl, 0.05 mM Na_2_HPO_4_, 0.15 mM KH_2_PO_4_, 11 mM glucose; containing a protease inhibitor cocktail, 1 g/100 ml (P8340, Sigma Chemical Co., www.sigmaaldrich.com) to remove hemocytes and other potential contaminants. In addition to midgut tissue, the following tissues were then excised, washed in PBS, immediately frozen on dry ice in a minimal amount of PBS, and stored at −80°C: fat body, Malpighian tubules, muscle, salivary glands, trachea, and ventral nerve cord. Just prior to analysis, the tissues were homogenized by hand with plastic pestles in 1.5-ml microcentrifuge tubes and centrifuged at 15,000 *g* for 15 min. Protein concentrations of the supernates were determined by the Coomassie Blue dye-binding method (Bio-Rad), using bovine serum albumin (BSA) as the standard. Triplicate replications of the supernates were applied to Costar high protein-binding ELISA plates (Fisher) at 500 ng/100 μl/well, using PBS, pH 7.4, as the diluent. The plates were blocked with 1% BSA in PBS, and then incubated sequentially with 200 ng/well of Mab 7D6, 1:2500 goat anti-mouse IgG-alkaline phosphatase conjugate (Sigma), and *p*-nitrophenylphosphate substrate (Pierce Chemical Co., www.piercenet.com). The plates were washed five times between steps with PBS containing 0.05% Tween 20. A standard curve was generated, using SigmaPlot software (Systat Software, Inc., www.systat.com), from two-fold dilutions of purified EF-1α assayed against Mab 7D6 on the same plate as the tissue homogenates. EF-1α used as the standard was purified from Sf21 cells as previously described (Stuart and Chamberlain 2003). Absorbances (A_410_) were read at 410 nm, and the EF-1α content of each well containing a tissue homogenate was calculated using the equation x = (y − b) ÷ m, where x = ng of EF-1α per well, y = A_410_ of the homogenate, b = the y-intercept, and m = the slope of the regression line generated for the standard curve. To express the EF-1α concentration as a percent of the total protein in a given tissue, the equation [(ng of EF-1α per well ÷ 500 ng of total protein per well) x 100] was used. EF-1α concentrations were compared among tissues by one-way analysis of variance (ANOVA), followed by the Tukey test to isolate differences in the mean concentrations.

### Detection of EF-1α in tissue homogenates by western blotting

Larval tissue homogenates originally prepared for the ELISA were stored at −80ºC until used for western blot analysis. The homogenates were rapidly thawed in a 37ºC waterbath and immediately diluted in SDS-PAGE sample buffer (0.0625 M Tris, pH 6.8, 2% SDS, 5% 2-mercaptoethanol, 20% glycerol, and 0.1% bromophenol blue) to a final concentration of 2 μg of protein/10 μl total volume/well. The samples were solubilized by incubation for 3 min in a boiling waterbath, cooled, and then loaded into the wells of denaturing 12% polyacrylamide mini-gels topped with 4% polyacrylamide stacking gels. After electrophoresis, the proteins were electroblotted onto polyvinylidene fluoride (PVDF) membranes (Fisher). Membranes were either stained with 0.1% Coomassie Blue R-250 in 40% methanol/10% acetic acid, or probed by western blot analysis. For western blotting, the membrane was blocked with 5% nonfat dry milk in Tris-buffered saline (TBS; 20 mM Tris-HCl, 500 mM NaCl, pH 7.5), and then probed by 60-min incubations in Mab 7D6 (1 μg/ml), followed by goat-anti-mouse IgG-alkaline phosphatase conjugate diluted 1:4000 in TBS containing 0.05% Tween 20 and 1% nonfat dry milk. The blot was washed extensively between steps in TBS-0.05% Tween 20. Development was allowed to ensue for 2 min in nitroblue tetrazolium/5-bromo-4-chloro-3-indolyl phosphate (NBT/BCIP) substrate solution (Bio-Rad). Staining intensities of immunoreactive proteins were compared between samples by reflectance densitometry (Tarlton and Knight 1996) using an Epson Model 4490 document scanner, and analyzed with ImageJ software (Rasband, W.S., ImageJ, U. S. National Institutes of Health, Bethesda, Maryland, USA, http://rsb.info.nih.gov/ij/, 1997-2006). Bio-Rad broad-range prestained standards served as molecular mass markers.

### Localization of EF-1α by immunofluorescence microscopy

Immunofluorescence microscopy was performed essentially as described previously (Habibi et al. 1998). In brief, thick sections were prepared from paraffin-embedded S. frugiperda second instars and mounted onto glass slides. The sections were deparaffinized and then hydrated through xylene, a graded alcohol series, and double-distilled water. Irrelevant protein binding sites on the slides were blocked by incubation with 3% bovine serum albumin overnight in a humidity chamber. The slides were probed with a 1:300 dilution of Mab 7D6, followed by goat anti-mouse IgG conjugated to Cy5 (Jackson ImmunoResearch Laboratories, www.jacksonimmuno.com). Negative control slides were processed in the same manner, except that Mab 7D6 and/or the Cy5-conjugated secondary antibody was omitted from the protocol. Both control and experimental sections were mounted in Mowiol and observed with a confocal laser scanning microscope (Bio-Rad). The images were visualized with CoMos software (Bio-Rad).

## Results and Discussion

**Figure 1:**
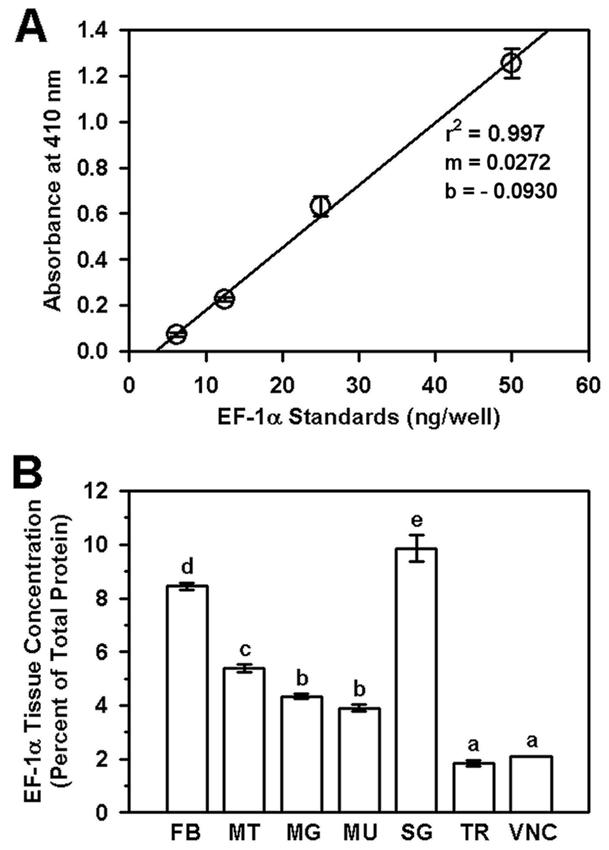
Measurement of EF-1α levels in larval tissues of Spodoptera frugiperda by ELISA. (A) A standard curve was generated by incubating Mab 7D6 with two-fold dilutions of purified EF-1α, plotted as ng/well versus absorbance at 410 nm. Data points (open circles) ± SD (error bars) and the resultant regression line (r^2^ = 0.997) are shown. Values for the slope (m) and y-intercept (b) of the line are given. (B) Tissue concentrations of EF-1α are presented as a percent of the total protein in each tissue (open bars) ± SD (error bars). Statistically different EF-1α concentrations are indicated by different letters (P<0.01, 1-way ANOVA and Tukey test). The tissues examined were fat body (FB), Malpighian tubules (MT), midgut (MG), muscle (MU), salivary gland (SG), trachea (TR), and ventral nerve cord (VNC).

This study revealed that EF-1α is an abundant constituent of all larval tissues of S. frugiperda (Fig. 1). The standard curve generated for purified EF-1α was linear between the concentrations of 6.25 ng/well and 50 ng/well (r^2^ = 0.997, Fig. 1A). Absorbance values obtained for the tissue homogenates fell within the linear portion of the standard curve. After calculating tissue concentrations of EF-1α from absorbance values, the enzyme was shown to comprise 1.9% to 9.9% of the total protein in the tissue homogenates (Fig. 1B).

The differences in EF-1α values may, to a certain extent, mirror the expected activity level of each of the represented tissues. The highest levels were found in the fat body (8.4%), which plays a major role in the overall metabolism of the insect (Chapman 1998), and the salivary gland (9.9%), which actively expresses numerous enzymes important for digestion (Musser et al. 2005; Parthasarathy and Gopinathan 2005). Moderate levels of EF-1α were seen in Malpighian tubules (5.4%), midgut (4.3%), and muscle (3.9%), tissues that play important roles in ion regulation, digestion and locomotion, respectively (Chapman 1998; Denholm and Skaer 2005; Terra and Ferreira 2005). These latter tissues expressed somewhat lower levels of EF-1α than the former tissues, possibly because of the timing within the instar at which they were analyzed as well as the fact that they may respond differently to endogenous factors such as juvenile hormone, known to modify EF-1α levels in some tissues (Zhou et al. 2002). The lowest levels of EF-1α were fouind in trachea and nerve cord. The trachea, in which EF-1α comprised 1.9% of the total protein, is primarily made up of single-layered epithelium cells, with its major function being to serve as a conduit for air (Uv and Samakovlis 2005). Thus, trachea would not be expected to have high levels of protein synthesis and therefore elongation factors. Few studies have looked at protein synthesis levels within the nervous system of insect larvae, (Klowden 2002). Therefore, it is hard to determine if our findings showing that the ventral nerve cord total protein only comprised 2.1% EF-1α is representative of nerve tissues (including the brain) from other species, or other instars within any given species. Overall, the EF-1α concentrations in the larval tissues were similar to those reported by Slobin (1980), who measured EF-1α levels in rabbit liver (5.3%), kidney (3.5%), brain (3.8%), and heart (1.0%) by radioimmunoassay. The highest concentration of EF-1α was found in uninduced Friend erythroleukemic cells (11.3%) (Slobin 1980), a cell line established from mouse hematopoietic cells infected by the Friend leukemia virus (Freedman and Lilly 1975).

**Figure 2:**
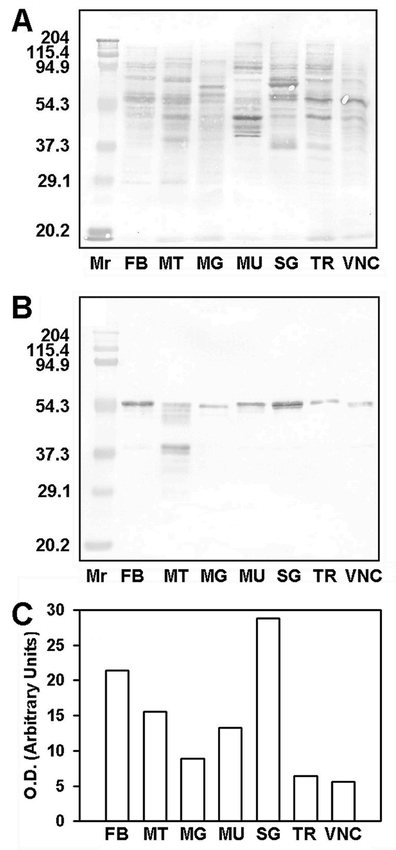
Western blot analysis of larval tissues from Spodoptera frugiperda fifth instars subjected to SDS-PAGE and transferred to PVDF membranes. (A) The membrane (5 μg of protein/lane) was stained with Coomassie Blue to visualize all proteins. Molecular mass markers (Mr) are expressed in kilodaltons. (B) The membrane (2 μg of protein/lane) was probed with 1 μg/ml Mab 7D6, followed by 1:4000 goat anti-mouse IgG-alkaline phosphatase conjugate and NBT/BCIP substrate, to visualize EF-1α. (C) Tissue concentrations of EF-1α shown in panel B were compared by reflectance densitometry. Relative optical densities (O.D.) are indicated by open bars. Tissue designations are as in Fig. 1.

Western blot analysis showed that EF-1α was present in all tissue homogenates as a protein of 57 kilodaltons (kDa) (Fig. 2B). This mass was slightly higher than the 53 kDa mass previously reported for S. frugiperda EF-1α (Stuart and Chamberlain 2003), presumably due to differences in the lots of prestained markers used as standards. A protein of 38 kDa was a minor reactive component in fat body and a major reactive component in Malpighian tubules. Additional immunoreactive components were seen in the Malpighian tubule homogenate at 27 kDa, 30–34 kDa, 36 kDa, and 51–57 kDa. It is likely that immunoreactive proteins smaller than 57 kDa are breakdown products of EF-1α rather than cross-reactive proteins, because prolonged storage of EF-1α initially purified to single-band homogeneity results in the appearance of immunoreactive bands similar in size to those seen in Fig. 2B (unpublished data). Why Malpighian tubules yielded a wider variety of immunoreactive EF-1α fragments than other tissues is unknown, given that all tissues received the same careful attention during preparation, i.e., the use of protease inhibitors, careful removal of the larval midgut prior to collection of other tissues, and maintenance of the homogenates on ice during handling. A comparison of relative EF-1α concentrations between tissues by western blotting and reflectance densitometry (Fig. 2C) generally followed the results obtained by ELISA (Fig. 1B).

**Figure 3:**
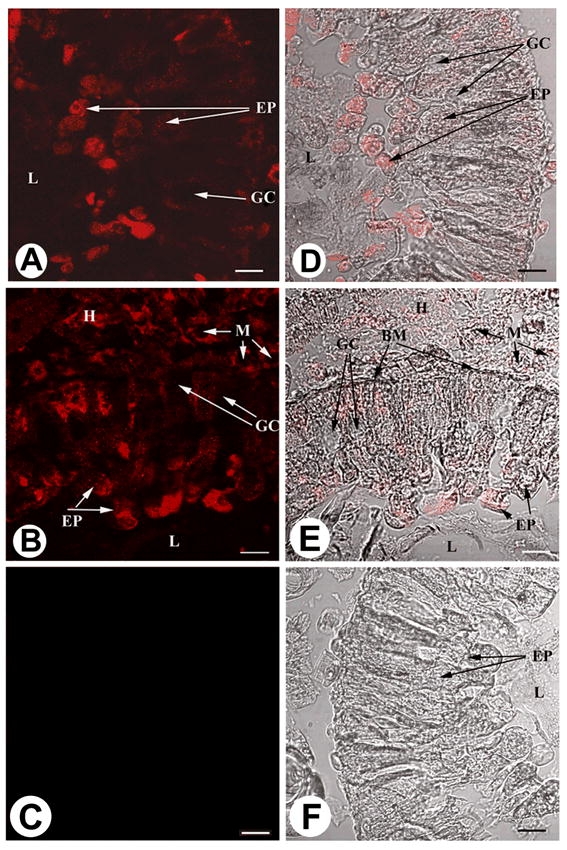
Immunofluorescence staining of paraffin-embedded thick sections of midgut tissue from the second instar Spodoptera frugiperda. Strong immunofluorescent signals were observed in panels A and D (anterior section), and B and E (posterior section) of the larval midgut, after incubation with Mab 7D6 (primary antibody), followed by goat anti-mouse IgG conjugated to Cy5 (secondary antibody). No immunofluorescent signals were observed in panels C and F, incubated with blocking agent and secondary antibody alone. Panels on the left are fluorescent images, while panels on the right are fluorescent images overlaid onto light microscopy images of the same sections. BM = Basement membrane, EP = epithelial cell, GC = goblet cell, H = hemolymph, L = lumen, M = Malpighian tubules. Scale bar = 20 μm.

Immunofluorescence microscopy of S. frugiperda second instars revealed variations in the amount of EF-1α expressed by different cell types within a given tissue (Fig. 3). Notably, the apical surfaces of midgut columnar epithelial cells yielded strong immunofluorescent signals, especially the brush-border microvilli, while goblet cells and basement membrane demonstrated no reactivity with Mab 7D6. Of particular interest is the expression of EF-1α within the larval midgut, because accumulation of EF-1α correlates with induction of apoptosis (Ruest et al. 2002; Schwientek et al. 2002; Duttaroy et al. 1998), and because apoptosis of the midgut epithelium is known to be an effective defense against baculovirus infection in the larval stages (Clem 2005). These observations have led us to speculate that the natural abundance of EF-1α in midgut epithelial cells might prime the cells for apoptosis following viral infection. It has been postulated that high levels of EF-1α enhance translation of pro-apoptotic factors such as caspase enzymes (Duttaroy et al. 1998), or cause distortion of the cell cytoskeleton by severing microtubules (Kato 1999), thus leading to cell death.

The absence of antibody staining in goblet cells was somewhat unexpected. While we cannot rule out the possibility that the EF-1α concentration in goblet cells was simply too low for detection using the method employed here, the absence of immunoreactivity may indicate the presence of an antigenically unique form, i.e. one encoded by a second gene not yet detected in lepidopterans, as suggested by Danforth and Ji (1998), or one created through post-translational modifications of the protein. Different isoforms of the EF-1α protein encoded by different genes are >90% homologous to one another within a given species (Mitchell et al. 1997,Cho et al. 1995). Nevertheless, the isoforms can be distinguished using monospecific antibodies, such as those raised to 42Sp50 in Xenopus laevis (Deschamps et al. 1991), or those generated to the mammalian EF-1α isoforms eEF1A1 and eEF1A2 (Khalyfa et al. 2001). Alternatively, EF-1α in goblet cells may be post-translationally modified to the extent that the protein is no longer recognized by Mab 7D6. We are currently examining lepidopteran EF-1α for the presence of post-translational modifications in order to measure their impact on recognition by Mab 7D6. Although further characterization of lepidopteran EF-1α remains to be done, the data in this paper support the hypothesis that EF-1α is differentially expressed in the larval tissues of S. frugiperda.

## Abbreviations

EF-1α: Elongation factor-1 alpha
Mab: Monoclonal antibody
ELISA: Enzyme-linked immunosorbent assay
